# Peripheral Blood Mononuclear Cell Transcriptomics in Porcine Reproductive and Respiratory Syndrome: A Window into Innate Immune Resistance and Tolerance to Viral Disease

**DOI:** 10.3390/v18090994

**Published:** 2026-09-09

**Authors:** Md Aminul Islam, Christiane Neuhoff, Maren Julia Pröll, Christine Große-Brinkhaus, Sharmin Aqter Rony, Ernst Tholen, Karl Schellander, Muhammad Jasim Uddin

**Affiliations:** 1 Department of Animal Breeding and Husbandry, Institute of Animal Science, University of Bonn, Endenicher Allee 15, 53115 Bonn, Germany; 2 Immunogenomics and Alternative Medicine Laboratory, Department of Medicine, Bangladesh Agricultural University, Mymensingh 2202, Bangladesh; 3 Department of Global Health and Social Medicine, Harvard Medical School, Boston, MA 02115, USA; 4 Department of Parasitology, Bangladesh Agricultural University, Mymensingh 2202, Bangladesh; 5 Virology and Infections Preparedness Laboratory, School of Veterinary Medicine, Murdoch University, Murdoch, WA 6150, Australia

**Keywords:** PBMCs, transcriptome, innate immunity, PRRSV, viral disease, host–pathogen interactions, resistance, tolerance, pig, gene editing

## Abstract

Peripheral blood mononuclear cells (PBMCs) are the most immunologically active and readily accessible fraction of whole blood, and they initiate host immune responses following infection and vaccination. Since PBMC transcriptomes capture diverse host–pathogen interactions, they offer a promising tool to uncover the genetic drivers of viral resistance and tolerance. As a detailed case study of this principle, we examine porcine reproductive and respiratory syndrome (PRRS), which remains one of the most economically important viral diseases of swine worldwide. Following PRRS virus (PRRSV) exposure, pigs rely on two complementary defense strategies: resistance, the capacity to limit viral replication, and tolerance, the capacity to sustain performance despite infection. Since resistance and tolerance phenotypes are difficult to measure directly through experimental challenge, indirect immune-trait measurements collected after vaccination offer a practical alternative. Most transcriptomic studies of the host response to PRRSV have focused on respiratory tissues, reflecting the virus’s tropism for pulmonary macrophages. However, intramuscularly delivered modified-live PRRSV vaccine reaches the bloodstream, bypassing the lung, so PBMCs, as the frontline defense system, mount the earliest measurable innate response. This review synthesizes the current literature on PBMC transcriptome models for deciphering innate resistance and tolerance to viral disease, using PRRS as our principal worked example; presents our own approach to profiling PBMCs after PRRSV vaccination; and outlines how the field has advanced since the original candidate-gene and QTL studies of the 2010s, including the recent FDA approval of the first CD163 gene-edited PRRSV-resistant pig line, and the emergence of single-cell and multi-tissue PBMC atlases, before considering how the same PBMC-based approach could extend to other host–virus interactions.

## 1. Introduction

Decades of selection for growth and reproduction have made modern pigs highly productive but not necessarily more resilient: animals bred intensively for output traits are often more susceptible to infection (lower resistance) or less able to sustain performance once infected (lower tolerance) [1]. Rising consumer concern over food safety, zoonotic risk, and animal welfare is adding pressure on breeders to control infectious diseases without relying solely on antibiotics [2,3,4]. Genetic control of disease offers durable advantages: better performance and product quality, reduced antimicrobial use, and improved welfare [2,3,4]. Host defense against infection operates through two complementary mechanisms: resistance, which lowers pathogen burden, and tolerance, which maintains performance despite a given pathogen burden [5]. Selecting for both traits is a pragmatic route to robust, productive herds even under continued infection pressure [6,7,8]. Yet, despite extensive phenotypic testing and advances in quantitative genetics, resistance and tolerance have remained difficult to capture as measurable molecular traits [9], underscoring the need for a more rational cell or tissue model for molecular phenotyping.

That need becomes more pronounced once host defense is broken down further. Host defense comprises innate immunity, the fast-acting, non-specific first line of defense that develops within hours and persists for days, and adaptive immunity, which is slower but pathogen-specific [10,11]. Since most RNA viruses, including PRRS virus (PRRSV), continually alter their antigenic epitopes, the less specific but rapid innate response is pivotal for controlling early infection [10]. Innate immune traits are attractive targets for selection precisely because they act quickly, protect broadly, and vary substantially between porcine breed lines [5,12,13]. Genes that shape the first hours of the innate response to PRRSV infection or vaccination are therefore strong candidates for resistance traits [14,15]. Live attenuated PRRSV vaccines induce an innate response in peripheral blood that mimics natural infection [16,17], and timely innate activation is a prerequisite for the adaptive response that ultimately clears the antigen [11].

This review has two aims: to synthesize what is currently known about PBMC transcriptomes as a model for dissecting the molecular-genetic basis of innate resistance and tolerance to viral disease, and to trace how that model has moved from single-candidate-gene hypotheses to genome-wide and now single-cell-resolved evidence, using host–PRRSV interactions as our detailed worked example throughout. We begin by describing PBMCs as a study system and positioning PBMC transcriptomics within the broader landscape of host–pathogen transcriptome profiling, followed by examining the specific molecular events through which PRRSV engages PBMCs and the tissue and cell models that have been used to characterize that engagement. We then turn to the concepts of resistance and tolerance themselves, reviewing the receptors, candidate genes, and quantitative trait loci (QTL) implicated in host response to PRRSV, and tracing how one of those candidates, CD163, has progressed from a receptor identified in cell culture to the basis of the first FDA-approved, PRRSV-resistant gene-edited pig line.

Building on that foundation, we present our own PBMC transcriptome profiling work following in vivo PRRSV vaccination, compare it with the direct-challenge alternative, and describe the breed-dependent variation it has revealed, before addressing the technical factors that govern the reliability of PBMC transcriptome data and the advantages, limitations, and recent methodological advances, including gene-expression deconvolution and single-cell sequencing, that bear on how confidently PBMC transcriptomes can be interpreted. We close by considering the prospects for this model as blood transcriptome annotation, genomic selection methods, and gene-editing technology continue to improve. Throughout, our central argument is that PBMCs, despite receiving comparatively less attention than organs or tissues in most viral disease research, offer a practical, welfare-friendly, and increasingly well-annotated window onto the molecular basis of innate resistance and tolerance to viral infection generally, a case we develop in detail for PRRS resistance and tolerance in pigs.

## 2. Search Strategy and Evidence Synthesis

This narrative, state-of-the-art synthesis intentionally bypasses strict PRISMA guidelines in favor of an integrative, cross-disciplinary approach to understanding porcine PBMC transcriptomes in PRRS resistance and tolerance [18,19]. Because this topic spans virology, innate immunology, quantitative genetics, functional genomics, gene editing, and regulatory science, a rigid systematic protocol or pooled-effect metric could not accommodate its scope. Instead, this review provides a representative, critically organized account of current knowledge without claiming exhaustive, auditable coverage of every publication [18,19].

To build this synthesis, PubMed/MEDLINE served as the primary database, supplemented by Scopus, Web of Science, Google Scholar, bioRxiv preprints, the FAANG data portal, CABI Agricultural and Bioscience resources, and the U.S. FDA Center for Veterinary Medicine’s Freedom of Information Summary regarding recent CD163 gene-edited pig approvals. Search queries combined concept blocks using Boolean logic across key parameters (sample/cell type, pathogen, host-response phenotype, technology, and translational biotechnology) through July 2026 without date restrictions (Supplementary Table S1). The additional literature was gathered via backward and forward citation chaining, prioritizing English-language, pig-focused sources while incorporating relevant human and murine findings to frame conserved signaling pathways like TLR/RIG-I-like receptors and JAK–STAT signaling. We prioritized including peer-reviewed research and reviews directly presenting PBMC or whole-blood transcriptomics, PRRSV host–pathogen interaction, or resistance/tolerance genetics, excluding data-poor conference abstracts and non-English sources. No formal risk-of-bias instrument was applied, consistent with this being an interpretive rather than a systematic synthesis; instead, findings are presented with enough methodological detail, species, tissue, challenge or vaccination design, and profiling platform, for readers to weigh their relevance directly, organized around the underlying biological pathway from viral entry through resistance/tolerance phenotypes and their translation into gene-edited animals.

## 3. PBMCs as a Study Tool

### 3.1. Composition and Isolation

**Figure 1:**
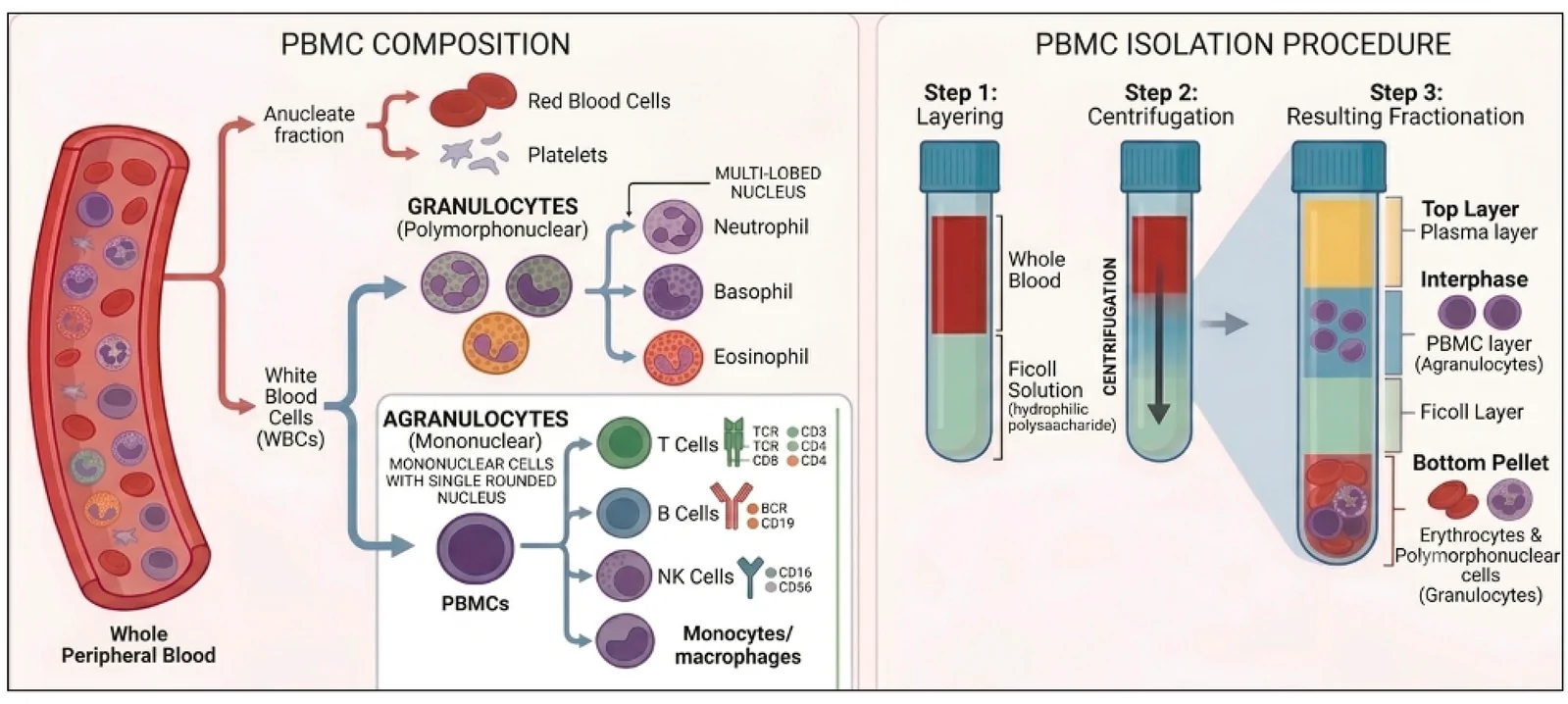
Composition and isolation methods of peripheral blood mononuclear cells (PBMCs). Schematic overview illustrating the distinct cell subsets present within PBMCs and the standard isolation technique used to harvest them.

PBMCs are peripheral blood cells with a single, rounded nucleus and are highly informative for the immune system of higher mammals [20,21,22]. Blood cells comprise anucleate components (red blood cells and platelets) and nucleated white blood cells (WBCs). WBCs fall into two main groups: polymorphonuclear granulocytes (neutrophils, basophils, eosinophils) and mononuclear agranulocytes, commonly known as PBMCs (Figure 1). PBMCs are readily isolated from whole blood via Ficoll density-gradient centrifugation, which separates blood components by specific gravity into a top plasma layer, a PBMC layer, and a bottom pellet of polymorphonuclear cells and erythrocytes [16].

### 3.2. Best Practice for Generating Robust PBMC Transcriptome Data

Extracting RNA from PBMCs isolated immediately after blood sampling yields the most robust transcriptome data. Key pre-analytical variables (including time to isolation, anticoagulant choice, storage temperature, cryopreservation, and plasma, granulocyte, or platelet contamination) can measurably alter PBMC transcriptomic profiles [23,24,25,26]. To minimize such variability, Mallone et al. [25] proposed standardized protocols across collection, isolation, shipping, cryopreservation, and thawing for T-cell assays. Furthermore, accurate normalization in RT-qPCR-based candidate gene studies requires validated reference genes. We previously evaluated nine candidate reference genes (B2M, BLM, GAPDH, HPRT1, PPIA, RPL4, SDHA, TBP, YWHAZ) in porcine PBMCs stimulated with LPS or LTA, and identified RPL4, PPIA, and B2M as the most suitable for this tissue [27,28].

### 3.3. Advances in Functional Annotation of PBMCs Transcriptomes

While the pig genome was assembled in 2010 [29], detailed annotation of its peripheral blood transcriptome and immunome has only recently advanced. The Functional Annotation of Animal Genomes (FAANG) initiative (www.faang.org) has established the core assays and data/metadata standards for porcine blood transcriptome studies [30]. A network-based expression atlas built from public porcine RNA-seq data has added substantial annotation depth across tissues, including blood [31]. Recently available high-quality annotations of the swine peripheral blood transcriptome [32] and multi-tissue immune atlas [33] establish a solid foundation for porcine PBMC transcriptomics.

### 3.4. Advantages of Using PBMC Model

Compared to invasive tissue sampling, PBMCs provide a low-stress, minimally invasive route for repeated longitudinal studies in the same animal. As circulating immune cells, PBMC transcriptomes capture both direct leukocyte function and systemic responses across distant organ systems [21,26,27,34,35]. These dual advantages (accessibility and broad biological representation) position PBMCs as a valuable model for biomarker discovery, disease monitoring, and host–pathogen research [21,35]. Consequently, PBMC transcriptomics enables rapid, welfare-friendly screening for viral resistance and tolerance in swine herds.

## 4. PBMC Transcriptomics as a Window into Host–Pathogen Interactions

Transcriptome profiling captures the complete set of RNA transcripts produced by a genome at a given time and has been widely used to elucidate the pathogenesis and genetics of host–pathogen interactions in pigs [36,37,38,39,40,41], in cattle [42], and in humans and non-human primates following vaccination [43]. Changes in gene expression underlie regulatory processes as diverse as nutrient absorption [44] and growth and metabolism [45]. As presented in Table 1, porcine PBMCs specifically have been profiled after in vitro lipopolysaccharide stimulation [46], in vivo Salmonella infection [47], Mycoplasma hyopneumoniae vaccination [48], and tetanus toxoid vaccination [26], and have proven useful for identifying immunocompetence traits and characterizing genetic variation in innate immune traits across pig populations [12,46,49]. Traits measured in PBMCs are heritable and genetically correlated with health and performance [50,51]. Global PBMC transcriptome studies have also shown pathogen-specific expression signatures, for example, in classical swine fever [52] and tetanus toxoid immunization [38]. In pigs infected with PRRSV in utero, PBMCs showed markedly increased IL-6, IL-10, and IFN-γ mRNA and an elevated IL-10/IL-12 ratio at both birth and 14 days of age relative to age-matched controls [53]. A recent multi-tissue transcriptomic atlas that profiled PBMCs alongside bone marrow, jejunum, lymph node, spleen, and thymus in Landrace pigs used deconvolution against sorted-cell references to track how PBMC composition and core immune gene expression shift with age and after poly(I:C) challenge, reinforcing PBMCs as a representative, minimally invasive readout of systemic innate immune status against viral and other challenges generally, not just PRRSV [33].

## 5. How PRRSV Engages the Porcine PBMC

PRRSV is the causative agent of one of the most economically damaging infectious diseases in the global swine industry, producing reproductive failure in breeding sows and respiratory disease in young and growing pigs [54,55]. It is a positive-sense, single-stranded RNA Arterivirus with two genetically divergent species, PRRSV-1 (European) and PRRSV-2 (North American)—formerly classified as type I and type II genotypes—that share roughly 70% nucleotide identity [6]. PRRSV replicates in the cytoplasm of cells of the porcine macrophage lineage and modulates host immunity from within that niche. Although natural infection targets pulmonary alveolar macrophages, intramuscularly delivered modified-live PRRSV vaccine reaches the blood directly, bypassing the lung, so PBMCs are the first cells to mount an innate response [18]. The degree to which a given vaccine strain itself infects and replicates in CD163^+^ cells varies with its level of attenuation, from early, more readily replicating modified-live strains to later, more attenuated strains that infect porcine CD163^+^ macrophages only poorly; vaccine response data are therefore a proxy for baseline innate immune kinetics rather than a substitute for characterizing the response to currently circulating field virus.

**Figure 2:**
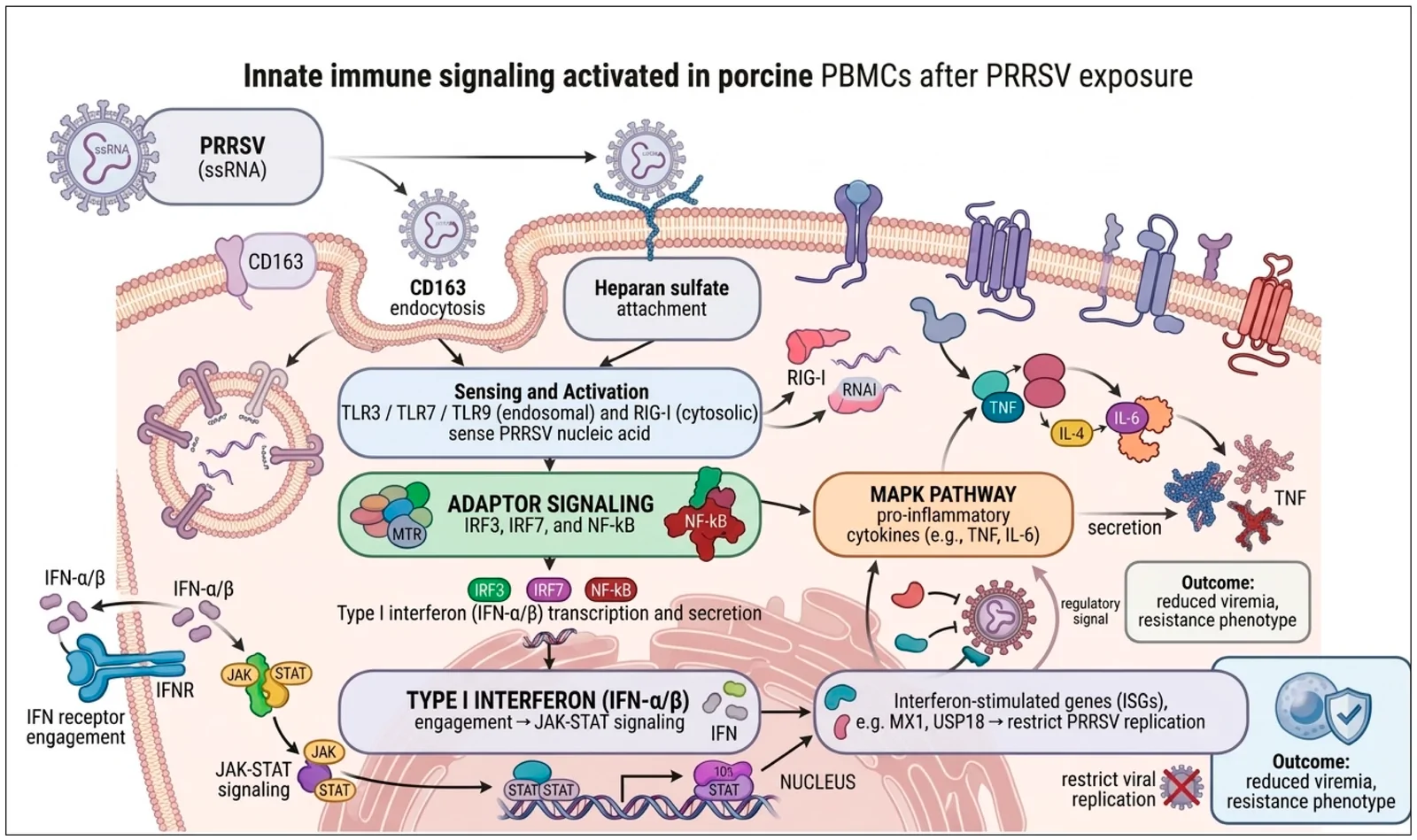
Activation of innate immune signaling in porcine PBMCs during PRRSV restriction. Schematic overview depicting PRRSV-induced activation of the innate immune response, moving sequentially through TLR/RIG-I recognition, IRF3/7 and NF-kB activation, type I interferon production, and downstream JAK–STAT signaling leading to interferon-stimulated gene (ISG) expression and viral suppression.

Like other mammals, the porcine innate response begins when pattern recognition receptors (PRRs) on host cells detect the conserved pathogen-associated molecular patterns (Figure 2). This signaling cascade is broadly conserved across viral infections and mammalian hosts, and PRRSV offers a well-characterized, economically important case through which to examine it in molecular detail. Toll-like receptors (TLRs), a major class of PRRs, are expressed on porcine PBMCs [56]; after vaccination, PRRSV nucleic acid is sensed by TLR3, TLR7, TLR9, and/or the RIG-I pathway [57,58]. Signaling through TLR and RIG-I-like receptor (RLR) adaptors activates the transcription factors IRF3, IRF7, and NF-κB, which together induce type I interferons (IFNs) and pro-inflammatory cytokines via the MAPK pathway [59]. Secreted IFN binds IFN receptors on neighboring cells and activates JAK–STAT signaling, inducing interferon-stimulated genes [60] whose protein products restrict PRRSV replication and help clear infection [61]. Lunney et al. [62] provide a detailed review of PRRSV pathogenesis and its interaction with host immunity elsewhere. Building on that foundation, we propose that transcriptome profiling across the course of the immune response is a rational strategy for uncovering the genetic basis of host–virus interaction generally, and we use host–PRRSV interaction here as our detailed illustrative case.

## 6. Tissue and Cell Models for Studying the Host–PRRSV Transcriptome

Because PRRSV shows marked tissue tropism, the choice of cells, tissue, or organ is critical to correctly identifying the genetic basis of the host transcriptional response. Table 2 summarizes the cell and tissue models most commonly used to characterize host–PRRSV transcriptome interactions: lung tissue, bronchial lymph node, pulmonary alveolar macrophages, uterine endothelium, dendritic cells, whole blood, and PBMCs. Lung tissue transcriptomes have been used to characterize the host immune response to PRRSV directly [55,63], and pulmonary alveolar macrophage transcriptomes have been profiled shortly after natural [64] and experimental [65,66,67] infection. Whole-blood transcriptomes have been analyzed with network-based approaches to identify pathway differences between PRRSV-susceptible and -resistant pigs [68,69,70]. To probe cytokine signaling in piglets with in utero PRRSV infection, constitutive mRNA expression was measured in PBMCs at birth and at 14 days of age and compared with age-matched controls [41]. Other PBMC studies of the transcriptional response to PRRSV [53,71] focused mainly on a small panel of candidate genes rather than the whole transcriptome. Whole-transcriptome profiling of porcine PBMCs after in vivo PRRSV vaccination therefore fills a gap, offering insights into host resistance and tolerance that candidate-gene approaches cannot provide alone [16,72,73].

Since these tissue and cell comparisons were established, single-cell resolution has become available for the respiratory compartment as well. A 2024 single-cell RNA-seq study of PRRSV- and African swine fever virus-infected porcine alveolar macrophages revealed a shared antiviral network (including PARP12, PARP14, HERC5, DDX60, RSAD2, and MNDA) that restricts replication of both viruses, highlighting single-cell resolution of cell-intrinsic responses masked by bulk transcriptomics [74]. Applying comparable single-cell or deconvolution methods to PBMCs is a natural next step for PRRS and other viral diseases alike (Section 8.5).

**Table 2:** Tissue/cell models used to characterize the host-PRRSV transcriptome interaction.

Tissue/Cell Model	Design and Technique	Purpose/Findings	Ref.
Lung tissue	In vivo, digital gene expression profiling	Pulmonary gene expression after highly virulent PRRSV (H-PRRSV) infection correlated with infection pathology; aggressive H-PRRSV replication and dissemination produced an aberrant host immune response.	[55,67,75]
Bronchial lymph node	In vivo, RNA-seq	Bronchial lymph node transcriptomes were profiled at 14 days post-infection.	[76]
Pulmonary alveolar macrophages (PAMs)	In vivo, microarray	Global transcriptome profiles explained differing susceptibility to PRRSV infection.	[75,77]
PAM	In vitro, RNA-seq	Transcriptome profiles 12 h post-infection with low- and high-virulence European PRRSV strains.	[68,78]
Uterine endothelium	In vivo, RNA-seq	Transcriptional response to PRRSV at the maternal–fetal interface and in the fetus after 21 days of experimental infection in pregnant gilts.	[79]
Dendritic cells	In vitro, microarray/RNA-seq	Transcriptome profiles of bone marrow-derived dendritic cells and monocytes after PRRSV/Streptococcus suis co-infection, and of lung dendritic cells from Duroc and Pietrain pigs.	[23,79]
Whole blood	In vivo, microarray/RNA-seq	Network-based analyses distinguished susceptible from resistant pigs across 0–42 dpi; blood transcriptomes at early time points correlated with weight gain and viral load phenotypes and with host-response QTL.	[68,69,70]
Whole blood	Vaccination challenge, RNA-seq, NanoString	Transcriptome profiles assessed the role of host genetics in PRRS vaccine efficacy and resistance/susceptibility to co-infection challenge, sampled at 4–21 days post-vaccination and 0–28 days post-challenge.	[80]
PBMCs	In utero infection, qRT-PCR	IL-6, IL-10, and IFN-γ mRNA were quantified in PBMCs of piglets infected in utero, at 0 and 14 days of age.	[53]
PBMCs	In vitro, qRT-PCR	CD28, CTLA-4, TGF-β, IL-2, and IL-10 mRNA were quantified after in vitro PRRSV or CSFV infection, with/without Astragalus polysaccharide.	[71]
PBMCs	Vaccination, microarray	Transcriptome profiles from pigs with extreme (high/low) PRRSV vaccine responses were compared with unvaccinated controls.	[72]
PBMCs	Vaccination, microarray	Transcriptomes from Landrace and Pietrain pigs were collected before and at 6, 24, 72 h and 28 days post primary vaccination with live attenuated PRRSV vaccine—the basis of our own model (Section 8).	[16,73]

## 7. Host Resistance and Tolerance: Concepts and Molecular Correlates

### 7.1. Defining Resistance and Tolerance

Following infection, a host either succumbs or survives; survivors either remain clinically healthy or experience illness followed by recovery. Two complementary defense mechanisms underlie survival: resistance, the ability to restrict or inhibit pathogen replication; and tolerance, the ability to maintain homeostasis and limit pathology despite a replicating pathogen [2,81]. Tolerance can be estimated as the slope of the regression of a host performance measure, growth rate, health status, and survival, on pathogen burden [82]; a fully tolerant pig’s health and productivity are unaffected by pathogen load. Tolerance mechanisms induced against one pathogen often confer some tolerance to unrelated pathogens as well [83], so selecting for tolerance is best implemented as part of an integrated herd health program that also manages pathogen exposure and environmental factors [84]. Although PRRS resistance has been extensively reviewed elsewhere [7], PRRS tolerance has received comparatively little attention, though Guy et al. [85] discussed both traits within the broader context of pig breeding. These concepts apply broadly to viral and non-viral livestock diseases. However, PRRS provides one of the best-characterized models for tracing their blood molecular correlates, which are crucial for managing infectious disease at the herd level.

### 7.2. Candidate Genes, Receptors, and QTL

Transcriptomic changes offer highly accessible and quantifiable molecular metrics for measuring resistance and tolerance [86]. Resistance-associated genes are thought to act either as barriers to PRRSV entry, reducing viremia, or as drivers of the active innate response to vaccination [14]. In infected pulmonary alveolar macrophages, overexpression of myxovirus resistance 1 (MX1) [87] and ubiquitin-specific protease 18 (USP18) [77] has been linked to reduced PRRSV replication. Beyond individual genes, quantitative trait loci (QTL) have also been associated with resistance and tolerance [88]; several groups, including ours [4,89], have mapped QTL for immune response, susceptibility, and resistance traits (reviewed in [84]). A genome-wide association study identified a major QTL on Sus scrofa chromosome 4 (SSC4) strongly associated with host response to PRRSV challenge [90,91], and single nucleotide polymorphisms in guanylate-binding protein 5 (GBP5) [92], GBP1 [93], and USP18 [94] each have been linked to PRRS resistance. Molecular signatures of tolerance are comparatively unexplored, but tolerance-associated genes are thought to suppress or limit the active cellular response to infection, or to protect the host from virus- or immune-mediated cytotoxicity without changing viremia [14,83,95]. Evidence-based major candidate genes, receptors, and QTL implicated in PRRS resistance and tolerance are summarized in Table 3.

Disease resistance can also stem from the presence or absence of cell-surface receptors required for pathogen entry [96]. Six candidate PRRSV receptors have already been described: the scavenger receptor CD163 [97], the cysteine-rich scavenger receptor SRCR [98], sialoadhesin (CD169/siglec-1) [99], CD151 [100], heparan sulfate [101], vimentin [102], and CD209 [103], as reviewed by Zhang and Yoo [104]. During natural infection, PRRSV attaches to alveolar macrophages via heparan sulfate glycosaminoglycans [105] and enters through CD163-mediated endocytosis [106]. Inside the early endosome, CD163 co-localizes with the virus [97], and the combination of CD163 engagement and endosomal acidification is thought to drive uncoating and genome release into the cytosol [97,107]. Besides alveolar macrophages, CD163, CD169, and CD28 are all expressed on PBMCs at various stages of activation and differentiation [21,22,46]. During viral entry, the vacuolar ATPase subunit ATP6V1B2 mediates the endosomal acidification required for viral uncoating [108]; because this acidification step depends on prior CD163-mediated internalization, the virus cannot be uncoated, replicate, or establish infection without functional CD163. Consequently, pigs lacking functional CD163 demonstrate strong resistance to PRRS.

**Table 3:** Candidate genes, receptors, and QTL implicated in PRRS resistance and tolerance.

Gene/Locus	Role in PRRSV Biology	Evidence	Ref.
CD163	Primary entry receptor; SRCR5 domain required for viral uncoating in early endosomes.	Receptor characterization; basis of first FDA-approved PRRSV-resistant gene-edited pig line (CD163Δexon7, approved April 2025).	[97,109,110,111,112,113,114,115]
SRCR	Candidate co-receptor supporting PRRSV attachment/entry.	In vitro receptor studies.	[98]
CD169 (Siglec-1, sialoadhesin)	Mediates monocyte/macrophage susceptibility to PRRSV depending on differentiation state.	In vitro infection studies.	[99]
CD151	Tetraspanin implicated in PRRSV entry.	Functional receptor studies.	[100]
Heparan sulfate	Initial attachment factor for PRRSV on macrophages.	Attachment/internalization studies.	[101,104]
Vimentin	Proposed co-receptor/cytoskeletal interaction partner.	Receptor-targeting studies.	[102]
CD209 (DC-SIGN)	C-type lectin implicated in PRRSV attachment.	Molecular cloning/binding studies.	[103]
ATP6V1B2	V-ATPase subunit; endosomal acidification required for PRRSV uncoating.	Functional characterization of V-ATPase.	[108]
MX1	Interferon-stimulated gene; overexpression associated with reduced PRRSV replication in PAMs.	PAM transcriptome studies.	[87]
USP18	ISG; restricts PRRSV replication; promoter SNP linked to resistance.	Functional and association studies.	[77,94]
GBP5	Interferon-inducible GTPase; candidate quantitative trait nucleotide for PRRSV host response.	GWAS/fine-mapping.	[92]
GBP1	Interferon-inducible GTPase; SNP associated with PRRSV resistance and growth performance.	Association study.	[93]
ADAM17	Downregulates CD163; higher vaccine-induced expression in German Landrace vs. Pietrain PBMCs, plausibly explaining relative resistance.	Our PBMC vaccination model.	[16,24,73,116]
SSC4 QTL (incl. WUR SNP)	Major QTL for host response to PRRSV challenge; also associated with longitudinal tolerance slope.	GWAS; genomic prediction; longitudinal tolerance analysis.	[90,91,117]
CD28	T-cell co-stimulatory molecule expressed on PBMCs; knockout increases IFN-γ response to PRRSV vaccination.	Gene-edited pig study.	[118]

### 7.3. From Candidate Gene to Commercial Gene-Edited Pig

CRISPR/Cas9 editing first demonstrated that pigs lacking CD163 are resistant to PRRSV challenge [109,110]. That original work targeted the SRCR5 domain of CD163 (encoded largely by exon 7), the region responsible for virus uncoating, while leaving the rest of the CD163 protein and its other physiological roles intact. Since then, multiple independent groups have reproduced and extended this result using different editing strategies and pig lines, including complete CD163 knockouts and precise exon 7/SRCR5 deletions, each yielding animals fully resistant to PRRSV-2 challenge [111,112,113,114]. Long-term follow-up of SRCR5-deleted pigs from birth to maturity found no differences in growth, health, meat composition, or reproductive performance relative to unedited animals, supporting the safety of this specific edit for commercial use [111]. Genus plc and its subsidiary Pig Improvement Company (PIC) subsequently introduced a single validated CD163 edit across four genetically diverse commercial lines and outlined the technical path to scaled, non-transgenic deployment of the resistance allele in breeding populations [119]. This effort culminated in April 2025, when the US FDA approved a CD163Δexon7 pig line as the first PRRSV-resistant, gene-edited food animal for commercial breeding and food use in the United States, based on challenge studies across multiple PRRSV isolates in which homozygous edited pigs consistently showed undetectable viral RNA and no seroconversion, while heterozygous and unedited controls did not [115]. This regulatory milestone converts a receptor identified through basic transcriptomic and virological research directly into a deployable resistance trait, and it strengthens the case for continuing to mine PBMC and other transcriptome data for the next generation of candidate targets.

It is worth noting that CD163-mediated resistance is context-dependent: CD163-null pigs remain susceptible to PRRSV-1 (European) PRRSV strains that utilize alternative entry residues [120] and are unprotected against unrelated pathogens like African swine fever virus (ASFV) [121], which instead requires RELA gene editing to confer resistance [122]. Separately, genetically modified pigs lacking CD28, a transcription factor and T-cell co-stimulatory molecule expressed on PBMCs, show heightened interferon-gamma activity, a potent antiviral innate response, after PRRSV vaccination [118]. Together, these findings argue for continuing to mine PBMC transcriptome profiles for genes and pathways that govern resistance and tolerance across a wider range of pathogens, not just PRRSV.

## 8. PBMC Transcriptome Models for Estimating PRRS Resistance and Tolerance

### 8.1. Direct Versus Indirect Phenotyping

Two broad experimental strategies exist for identifying candidate genes for resistance and tolerance: the direct infection/disease model and the indirect immunization (vaccination) model. Under the direct model, resistance is typically quantified by infection severity (e.g., viral load), and tolerance by changes in performance, growth rate, feed intake, and litter size relative to pathogen burden [123]. Direct phenotyping requires individual animal identification, disease registration, and a challenge protocol that is difficult to implement in intensive commercial production [5], and deliberately selecting for resistance/tolerance to one pathogen can increase susceptibility to others [124]. The indirect model instead measures transcriptome alterations after vaccination with an avirulent PRRSV strain, and it also supports evaluating tolerance against multiple stimuli (pathogens) simultaneously. Vaccination does not cause illness, but it does prompt the immune system to generate memory T cells, B cells, and antibodies [17], and a genomic marker of vaccine-induced immunocompetence, heritable and correlated with health and performance [6,49], offers a more practical, welfare-friendly route to the same information. A broader, innate-response-focused version of this strategy is likely to generalize better across a wider range of viral pathogens beyond PRRSV, wherever PBMC access is similarly convenient [12].

### 8.2. From Candidate Genes to Whole-Transcriptome Scans

Two complementary strategies exist for quantifying the gene expression changes that underlie resistance and tolerance: the candidate gene approach and the whole-genome/whole-transcriptome scan [125]. Candidate gene studies depend on prior knowledge of the physiological, biochemical, or functional role of the gene in question and struggle to capture multiple genes acting jointly on a phenotype [126]. Whole-transcriptome profiling instead provides a genome-wide picture of which genes are active during infection or vaccination and has proven a productive starting point for identifying candidates for host immunocompetence, susceptibility, and resistance [127,128]. While candidate gene studies are useful for elucidating the specific functions of individual genes, whole-genome profiling provides a broader perspective by capturing the coordinated action of multiple genes and pathways, reflecting the polygenic nature of host immune responses.

### 8.3. Our PBMC Vaccination Model for Time-Course Investigation

Genomic selection for disease tolerance is more complex than for resistance, as tolerance reflects dynamic changes in performance relative to pathogen burden over time rather than a single point measurement. We applied a PBMC transcriptome model to estimate temporal changes in host immunocompetence following in vivo PRRSV vaccination in pigs [16]. Within 72 h of vaccination, we observed a substantial transcriptome shift in PBMCs, involving roughly 3000 differentially expressed genes, and weighted network analysis of the significantly altered transcripts yielded a focused list of candidate genes for host immunocompetence. Because it mimics infection without its hazards, the in vivo vaccination model is both less risky and more animal-welfare-friendly than natural or experimental challenge [6,17]. Our results captured the variation in early-stage PBMC transcriptional response to PRRSV vaccination in German Landrace (DL) [16] and Pietrain (Pi) pigs [73], and we found substantial differences in PBMC transcriptome profiles between vaccinated DL and Pi pigs [116], consistent with hub genes from the PBMC-derived functional network representing plausible candidates for PRRS resistance and tolerance more broadly. It is worth emphasizing that vaccine-strain response data are a proxy for baseline innate immune kinetics, not a substitute for characterizing the response to currently circulating field virus: many modified-live strains, including those underlying our own model, derive from isolates that are now decades old, and contemporary field strains have continued to evolve substantially since. Extending this PBMC framework to actively circulating isolates, where feasible, remains an important next step.

Differential expression peaked sharply: a total of 542, 2263, and 357 genes were differentially expressed at 6, 24, and 72 h post-vaccination relative to unvaccinated controls, with the response reaching its magnitude peak at 24 h, the apparent optimal window for capturing the innate transcriptome response [16]. The genes altered by vaccination overlapped substantially with known immune response genes and genes previously implicated in PRRSV infection [55,67,78,87,129], supporting PBMCs as a promising tissue for uncovering resistance and tolerance signatures.

Modeling viremia trajectories during PRRSV infection showed that the resistance-associated WUR SNP also correlates with overall tolerance slope, indicating that simultaneous trait selection is feasible but depends on repeated-measures infection or vaccination data rather than single-time-point phenotyping [116]. PBMC transcriptome time courses of the kind used in our own vaccination studies [16,73,116] are well suited to generating exactly this kind of repeated-measures data non-invasively.

### 8.4. Breed-Dependent Variation

Natural variation in PRRSV resistance and tolerance across swine breeds [75,130,131] supports using marker-assisted selection for PRRS control [132]. This variation has been documented across several cell and tissue models: PRRSV-induced transcriptomes of lung dendritic cells differ between Duroc and Pietrain pigs [23], and transcriptomes of in vitro PRRSV-infected pulmonary alveolar macrophages differ between Landrace and Pietrain pigs [75]. Relative susceptibility across breeds has been ranked, from most to least susceptible, as Hampshire > Large White > Duroc > Landrace (reviewed in [84]). We likewise found considerable differences in PBMC transcriptome profiles between vaccinated DL and Pi pigs [116], most notably a significant overexpression of ADAM17 (a disintegrin and metalloprotease domain 17) in DL PBMCs relative to Pi. ADAM17 downregulates CD163 [24], the receptor that facilitates PRRSV endocytosis into macrophages, so the higher vaccine-induced ADAM17 expression in DL pigs offers a plausible molecular explanation for their relatively greater PRRS resistance. This finding illustrates how PBMC transcriptome profiling can resolve breed-level variations in PRRSV resistance or tolerance, further supporting the PBMC model as a viable tool for evaluating host response to infection.

### 8.5. PBMC Cell-Type Heterogeneity and How It Is Now Being Addressed

The primary limitation of the PBMC model is its inability to map transcriptomic changes to specific cell types on its own [133,134]. Because PBMCs comprise a heterogeneous mix of monocytes, T cells, B cells, natural killer cells, and dendritic cells, shifts in cell composition can easily be confounded with changes in per-cell gene expression [135,136]. Moreover, Cell-type-specific expression profiling in pigs has historically been limited by a scarcity of validated immune-cell antibodies (reviewed in [127]). Computational deconvolution offers a practical workaround: gene expression deconvolution [137] and cell-type enrichment analysis [138] estimate cell-type composition from bulk data, utilizing statistical approaches reviewed by Zhao and Simmons [139]. Most deconvolution methods rely on the linear mixture framework introduced by Venet et al. [140], which models a sample’s gene expression as a composition-weighted average of pure cell-type profiles, where cell proportions remain constant across all genes but vary between samples.

The deconvolution approach has matured substantially. A 2025 study established reference profiles for eight sorted porcine PBMC subpopulations (including T, B, NK, and myeloid cells) and used CIBERSORTx, a machine-learning method, to deconvolve bulk PBMC data across developmental stages and poly(I:C) stimulation [32], directly addressing the cell heterogeneity limitations of bulk transcriptomics. Single-cell RNA-seq provides a complementary, reference-free alternative: a 2024 scRNA-seq study of PRRSV- and ASFV-infected porcine alveolar macrophages unmasked cell-intrinsic antiviral programs diluted in bulk sequencing [74], supported by new pig-specific single-cell immune atlases for cell-type annotation [141]. Combining PBMC vaccination time-course designs with deconvolution or single-cell sequencing represents a logical next step to pinpoint which subpopulations drive specific resistance or tolerance signals in porcine innate antiviral immunity.

## 9. Discussions

**Figure 3:**
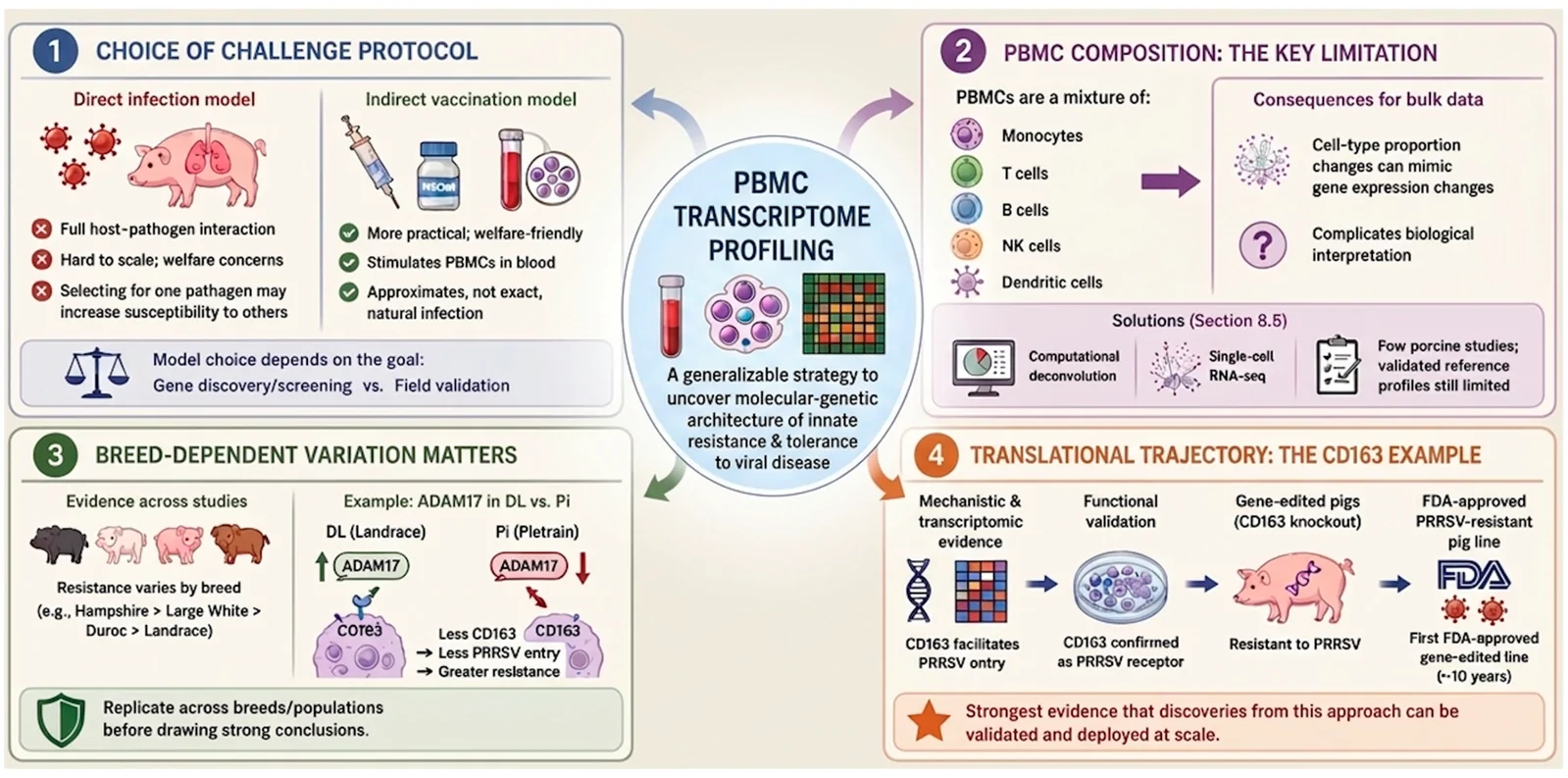
PBMC transcriptome profiling as a framework for assessing innate disease resistance and tolerance. Schematic representation of PBMC transcriptomic profiling, using porcine reproductive and respiratory syndrome as a model system. The four thematic areas critical for evaluating generalizability across other viral disease models are highlighted.

Taken together, the evidence reviewed here supports a central claim of this review: PBMC transcriptome profiling is a generalizable strategy for deciphering the molecular-genetic architecture of innate resistance and tolerance to viral disease, and PRRS provides the most complete existing test of that strategy in a single, well-studied host–pathogen system. Whether this framework extends beyond PRRS, however, turns on four methodological and translational considerations, illustrated in Figure 3, which we weigh in turn below.

The choice of challenge protocol materially affects what a given study can detect. Direct infection followed by phenotyping captures the full host–pathogen interaction but is difficult to scale in commercial systems and raises welfare concerns [5], and deliberately selecting for resistance or tolerance to one pathogen can increase susceptibility to others [124]. The indirect vaccination model that we and others have used [16,73,116] is more tractable and welfare-friendly, since intramuscular vaccine antigen contacts blood immune cells, PBMCs, without ever reaching the pulmonary alveolar macrophages that natural infection targets first; but it only approximates, rather than reproduces, the innate response to virulent challenge [6,17]. Which model is preferable depends on the translational goal rather than one approach being uniformly superior: the vaccination model is the more efficient screen for candidate genes, while direct challenge remains the standard for validating resistance under field conditions, and a mature research program should draw on both, in that order.

PBMC composition is arguably the model’s most consequential limitation. Because PBMCs are a mixture of monocytes, T cells, B cells, NK cells, and dendritic cells, bulk transcriptome shifts can reflect changes in cell-type proportions as readily as changes in per-cell gene expression [133,134,135,136], which complicates the biological interpretation of any candidate gene identified from bulk data. The deconvolution and single-cell approaches discussed in Section 8.5 [32,74,141] directly address this limitation, but they have so far been applied in only a handful of porcine studies, and standardized, validated PBMC-subtype reference profiles remain comparatively scarce next to those available for humans and mice.

The evidence for breed-dependent variation in PRRSV resistance and tolerance [23,24,75,84,116], including our own finding of differential ADAM17 expression between German Landrace and Pietrain pigs [24,116], points to a further complication: candidate genes and pathways identified in one breed or population may not transfer directly to another. Replication across genetic backgrounds strengthens confidence in any candidate gene, though the animal numbers required for adequately powered replication across multiple breeds are substantial and rarely feasible within a single study; multi-institutional consortia or meta-analytic pooling of smaller breed-specific cohorts offer a more tractable path toward this goal than expecting single-study replication.

Set against these caveats, the translational trajectory of CD163 is the strongest available evidence that the underlying research program is target-oriented rather than merely descriptive. CD163 moved from a receptor implicated through mechanistic and transcriptomic studies of PRRSV entry to the basis of the first FDA-approved, gene-edited PRRSV-resistant pig line within roughly a decade [100,104,105]. That translational arc did not depend on PBMC transcriptomics specifically, but it demonstrates that candidate genes surfaced through this general class of approaches can be validated and deployed at scale, the strongest argument available for extending PBMC transcriptome profiling, carefully and with the limitations above in view, to other viral diseases of livestock, companion animals, and humans where the molecular-genetic basis of innate resistance and tolerance remains incompletely understood. For researchers deciding whether to adopt this framework, the practical implication is straightforward: PBMC transcriptomics is best deployed as a first-pass discovery tool, and any candidate gene it surfaces should be expected to require breed replication, cell-resolved validation, and testing against contemporary field-relevant challenge before it can inform a resistance-breeding or gene-editing program.

## 10. Conclusions

PBMC transcriptome profiling offers a minimally invasive, repeatable window into the molecular-genetic basis of innate resistance and tolerance to viral disease, with PRRS serving here as a detailed, well-evidenced case study of that broader principle. Across the studies reviewed, PBMC-based approaches have progressed from single-candidate-gene analyses to genome-wide and, increasingly, single-cell-resolved profiling, culminating in the translation of CD163 from a mechanistically characterized receptor to the first FDA-approved, gene-edited PRRSV-resistant pig line; the breed-dependent transcriptional differences identified using this model further support its value for candidate discovery. At the same time, the approach has limitations: cellular heterogeneity complicates interpretation of bulk transcriptome data, and vaccination-based profiling approximates rather than reproduces the response to virulent field virus, so findings from this model should be treated as hypothesis-generating rather than conclusive pending validation with cell-resolved and field-strain data. Extending PBMC transcriptome profiling to pre- and post-challenge designs, paired with deconvolution or single-cell methods, is a logical next step for resolving these limitations, for PRRS and for the many other viral diseases where the molecular-genetic basis of innate resistance and tolerance remains incompletely understood.

## Figures and Tables

**Table 1:** Studies using porcine PBMC transcriptomes to decipher host immune-transcriptomic responses.

Disease/Pathogen Model	Study Design and Major Findings	Ref.
Clinically healthy pigs	PBMC transcriptomes were examined to estimate heritability of a wide range of immune traits, and the relationship between immune traits and performance was assessed in both specific-pathogen-free (SPF) and non-SPF environments.	[49]
Generalized immune stimulation (*M. hyopneumoniae* vaccination)	PBMC transcriptomes were profiled at three weeks post-vaccination. Heritability was estimated for 54 immune traits (antibodies, inflammatory proteins, cell subsets, ex vivo cytokine production, phagocytosis, lymphocyte proliferation); a total of 30 of 54 traits were highly heritable (h^2^ > 0.4).	[12,36]
Concanavalin A (Con A)	Microarray-based PBMC transcriptome profiling identified 46, 452, and 418 differentially expressed genes at 3, 20, and 68 h post in vitro Con A stimulation.	[37]
Con A	Mitogen-stimulated PBMCs were studied for phagocytic activity and virus-induced IFN-α production to estimate genetic variation in immune traits.	[13]
Poly(I:C)	RNA-seq-based PBMC transcriptome profiling compared Dapulian (Chinese indigenous breed) and Landrace pigs stimulated with poly(I:C).	[41]
Lipopolysaccharide (LPS)/PMA	Global transcriptome profiles 24 h after in vitro LPS and PMA stimulation established PBMCs as a suitable tool for studying immunity and disease resistance.	[46]
Tetanus toxoid (model antigen)	Microarray-based PBMC transcriptomes were compared before and after primary and booster vaccination; a total of 679 genes were differentially expressed at 2 h post-vaccination, mapping to 110 canonical immune pathways.	[38]
Tetanus toxoid (continuation)	PBMC transcriptome responses were linked to lean growth and humoral immune performance phenotypes.	[39]
Tetanus toxoid (continuation)	PBMC transcriptomic responses were compared between primary and secondary vaccination against a background of extreme lean growth and antibody titers.	[40]
*Salmonella enterica* serovar Typhimurium	Microarray-based transcriptional changes in PBMCs were measured in response to Salmonella inoculation at 0 and 2 days post-inoculation (dpi).	[47]
*Mycoplasma hyopneumoniae*	Microarray-based PBMC transcriptome profiling at two weeks post-vaccination identified biomarkers for phagocytosis and CD4^−^/CD8^+^ cell-count phenotypes.	[48]
Classical swine fever virus	Microarray-based PBMC transcriptome profiling of a highly virulent CSFV strain (Shimen) showed 54–438 genes upregulated and 61–218 genes downregulated across 1, 3, 6, and 9 dpi.	[52]

## Data Availability

No new data were created in this study. All data discussed are available in the cited publications or from the corresponding author upon reasonable request.

## Supplementary Materials

The following supporting information can be downloaded at: https://www.mdpi.com/article/10.3390/v18090994/s1, Supplementary Table S1: Concept blocks and Boolean search combinations used for literature retrieval.

## References

[1] Prunier A., Heinonen M., Quesnel H. (2010). High Physiological Demands in Intensively Raised Pigs: Impact on Health and Welfare. Anim. Int. J. Anim. Biosci..

[2] Stear M.J., Bishop S.C., Mallard B.A., Raadsma H. (2001). The Sustainability, Feasibility and Desirability of Breeding Livestock for Disease Resistance. Res. Vet. Sci..

[3] Visscher A.H., Janss L.L.G., Niewold T.A., de Greef K.H. (2002). Disease Incidence and Immunological Traits for the Selection of Healthy Pigs. A Review. Vet. Q..

[4] Uddin M.J., Cinar M.U., Grosse-Brinkhaus C., Tesfaye D., Tholen E., Juengst H., Looft C., Wimmers K., Phatsara C., Schellander K. (2011). Mapping Quantitative Trait Loci for Innate Immune Response in the Pig. Int. J. Immunogenet..

[5] Doeschl-Wilson A.B., Bishop S.C., Kyriazakis I., Villanueva B. (2012). Novel Methods for Quantifying Individual Host Response to Infectious Pathogens for Genetic Analyses. Front. Genet..

[6] Rowland R.R.R., Lunney J., Dekkers J. (2012). Control of Porcine Reproductive and Respiratory Syndrome (PRRS) through Genetic Improvements in Disease Resistance and Tolerance. Front. Genet..

[7] Reiner G. (2016). Genetic Resistance—An Alternative for Controlling PRRS?. Porc. Health Manag..

[8] Mulder H.A., Rashidi H. (2017). Selection on Resilience Improves Disease Resistance and Tolerance to Infections. J. Anim. Sci..

[9] Glass E.J., Baxter R., Leach R.J., Jann O.C. (2012). Genes Controlling Vaccine Responses and Disease Resistance to Respiratory Viral Pathogens in Cattle. Vet. Immunol. Immunopathol..

[10] Beutler B. (2004). Innate Immunity: An Overview. Mol. Immunol..

[11] Beirag N., Varghese P.M., Kishore U. (2025). Innate Immune Response to Viral Infection. Adv. Exp. Med. Biol..

[12] Flori L., Gao Y., Laloë D., Lemonnier G., Leplat J.-J., Teillaud A., Cossalter A.-M., Laffitte J., Pinton P., de Vaureix C. (2011). Immunity Traits in Pigs: Substantial Genetic Variation and Limited Covariation. PLoS ONE.

[13] Edfors-Lilja I., Wattrang E., Magnusson U., Fossum C. (1994). Genetic Variation in Parameters Reflecting Immune Competence of Swine. Vet. Immunol. Immunopathol..

[14] Glass E.J. (2012). The Molecular Pathways Underlying Host Resistance and Tolerance to Pathogens. Front. Genet..

[15] Loving C.L., Osorio F.A., Murtaugh M.P., Zuckermann F.A. (2015). Innate and Adaptive Immunity against Porcine Reproductive and Respiratory Syndrome Virus. Vet. Immunol. Immunopathol..

[16] Islam M.A., Große-Brinkhaus C., Pröll M.J., Uddin M.J., Rony S.A., Tesfaye D., Tholen E., Hölker M., Schellander K., Neuhoff C. (2016). Deciphering Transcriptome Profiles of Peripheral Blood Mononuclear Cells in Response to PRRSV Vaccination in Pigs. BMC Genom..

[17] Siegrist C.-A. (2012). Vaccine: General Aspects of Vaccinations.

[18] Grant M.J., Booth A. (2009). A Typology of Reviews: An Analysis of 14 Review Types and Associated Methodologies. Health Inf. Libr. J..

[19] Barry E.S., Merkebu J., Varpio L. (2022). Understanding State-of-the-Art Literature Reviews. J. Grad. Med. Educ..

[20] Fan H., Hegde P.S. (2005). The Transcriptome in Blood: Challenges and Solutions for Robust Expression Profiling. Curr. Mol. Med..

[21] Mohr S., Liew C.-C. (2007). The Peripheral-Blood Transcriptome: New Insights into Disease and Risk Assessment. Trends Mol. Med..

[22] Letzkus M., Luesink E., Starck-Schwertz S., Bigaud M., Mirza F., Hartmann N., Gerstmayer B., Janssen U., Scherer A., Schumacher M.M. (2014). Gene Expression Profiling of Immunomagnetically Separated Cells Directly from Stabilized Whole Blood for Multicenter Clinical Trials. Clin. Transl. Med..

[23] Pröll M.J., Neuhoff C., Schellander K., Uddin M.J., Cinar M.U., Sahadevan S., Qu X., Islam M.A., Poirier M., Müller M.A. (2017). Transcriptome Profile of Lung Dendritic Cells after In Vitro Porcine Reproductive and Respiratory Syndrome Virus (PRRSV) Infection. PLoS ONE.

[24] Guo L., Niu J., Yu H., Gu W., Li R., Luo X., Huang M., Tian Z., Feng L., Wang Y. (2014). Modulation of CD163 Expression by Metalloprotease ADAM17 Regulates Porcine Reproductive and Respiratory Syndrome Virus Entry. J. Virol..

[25] Mallone R., Mannering S.I., Brooks-Worrell B.M., Durinovic-Belló I., Cilio C.M., Wong F.S., Schloot N.C. (2011). Isolation and Preservation of Peripheral Blood Mononuclear Cells for Analysis of Islet Antigen-Reactive T Cell Responses: Position Statement of the T-Cell Workshop Committee of the Immunology of Diabetes Society. Clin. Exp. Immunol..

[26] Yang J., Diaz N., Adelsberger J., Zhou X., Stevens R., Rupert A., Metcalf J.A., Baseler M., Barbon C., Imamichi T. (2016). The Effects of Storage Temperature on PBMC Gene Expression. BMC Immunol..

[27] Cinar M.U., Islam M.A., Pröll M., Kocamis H., Tholen E., Tesfaye D., Looft C., Schellander K., Uddin M.J. (2013). Evaluation of Suitable Reference Genes for Gene Expression Studies in Porcine PBMCs in Response to LPS and LTA. BMC Res. Notes.

[28] Uddin M.J., Cinar M.U., Tesfaye D., Looft C., Tholen E., Schellander K. (2011). Age-Related Changes in Relative Expression Stability of Commonly Used Housekeeping Genes in Selected Porcine Tissues. BMC Res. Notes.

[29] Archibald A.L., Bolund L., Churcher C., Fredholm M., Groenen M.A.M., Harlizius B., Lee K.-T., Milan D., Rogers J., Rothschild M.F. (2010). Pig Genome Sequence—Analysis and Publication Strategy. BMC Genom..

[30] Tuggle C.K., Giuffra E., White S.N., Clarke L., Zhou H., Ross P.J., Acloque H., Reecy J.M., Archibald A., Bellone R.R. (2016). GO-FAANG Meeting: A Gathering On Functional Annotation of Animal Genomes. Anim. Genet..

[31] Summers K.M., Bush S.J., Wu C., Su A.I., Muriuki C., Clark E.L., Finlayson H.A., Eory L., Waddell L.A., Talbot R. (2019). Functional Annotation of the Transcriptome of the Pig, Sus Scrofa, Based Upon Network Analysis of an RNAseq Transcriptional Atlas. Front. Genet..

[32] Liu H., Smith T.P.L., Nonneman D.J., Dekkers J.C.M., Tuggle C.K. (2017). A High-Quality Annotated Transcriptome of Swine Peripheral Blood. BMC Genom..

[33] Zhao Q., Wang J., Ma F., Chen Q., Liu H., Yang J., Chen S., Tang Y., Mi S., Wang L. (2025). The Comprehensive Transcriptomic Atlas of Porcine Immune Tissues and the Peripheral Blood Mononuclear Cell (PBMC) Immune Dynamics Reveal Core Immune Genes. J. Anim. Sci. Biotechnol..

[34] Park K.-D., Park J., Ko J., Kim B.C., Kim H.-S., Ahn K., Do K.-T., Choi H., Kim H.-M., Song S. (2012). Whole Transcriptome Analyses of Six Thoroughbred Horses before and after Exercise Using RNA-Seq. BMC Genom..

[35] Chaussabel D. (2015). Assessment of Immune Status Using Blood Transcriptomics and Potential Implications for Global Health. Semin. Immunol..

[36] Flori L., Gao Y., Oswald I.P., Lefevre F., Bouffaud M., Mercat M.-J., Bidanel J.-P., Rogel-Gaillard C. (2011). Deciphering the Genetic Control of Innate and Adaptive Immune Responses in Pig: A Combined Genetic and Genomic Study. BMC Proc..

[37] Wilkinson J.M., Dyck M.K., Dixon W.T., Foxcroft G.R., Dhakal S., Harding J.C. (2012). Transcriptomic Analysis Identifies Candidate Genes and Functional Networks Controlling the Response of Porcine Peripheral Blood Mononuclear Cells to Mitogenic Stimulation. J. Anim. Sci..

[38] Adler M., Murani E., Brunner R., Ponsuksili S., Wimmers K. (2013). Transcriptomic Response of Porcine PBMCs to Vaccination with Tetanus Toxoid as a Model Antigen. PLoS ONE.

[39] Adler M., Murani E., Ponsuksili S., Wimmers K. (2013). PBMC Transcription Profiles of Pigs with Divergent Humoral Immune Responses and Lean Growth Performance. Int. J. Biol. Sci..

[40] Adler M., Murani E., Ponsuksili S., Wimmers K. (2015). PBMC Transcriptomic Responses to Primary and Secondary Vaccination Differ Due to Divergent Lean Growth and Antibody Titers in a Pig Model. Physiol. Genom..

[41] Wang J., Wang Y., Wang H., Wang H., Liu J.-F., Wu Y., Guo J. (2016). Transcriptomic Analysis Identifies Candidate Genes and Gene Sets Controlling the Response of Porcine Peripheral Blood Mononuclear Cells to Poly I:C Stimulation. G3 Genes Genomes Genet..

[42] Blanco F.C., Soria M., Bianco M.V., Bigi F. (2012). Transcriptional Response of Peripheral Blood Mononuclear Cells from Cattle Infected with *Mycobacterium bovis*. PLoS ONE.

[43] Wang I.-M., Bett A.J., Cristescu R., Loboda A., Ter Meulen J. (2012). Transcriptional Profiling of Vaccine-Induced Immune Responses in Humans and Non-Human Primates. Microb. Biotechnol..

[44] de Mello V.D.F., Kolehmanien M., Schwab U., Pulkkinen L., Uusitupa M. (2012). Gene Expression of Peripheral Blood Mononuclear Cells as a Tool in Dietary Intervention Studies: What Do We Know So Far?. Mol. Nutr. Food Res..

[45] Ayuso M., Fernández A., Núñez Y., Benítez R., Isabel B., Barragán C., Fernández A.I., Rey A.I., Medrano J.F., Cánovas Á. (2015). Comparative Analysis of Muscle Transcriptome between Pig Genotypes Identifies Genes and Regulatory Mechanisms Associated to Growth, Fatness and Metabolism. PLoS ONE.

[46] Gao Y., Flori L., Lecardonnel J., Esquerré D., Hu Z.-L., Teillaud A., Lemonnier G., Lefèvre F., Oswald I.P., Rogel-Gaillard C. (2010). Transcriptome Analysis of Porcine PBMCs after In Vitro Stimulation by LPS or PMA/Ionomycin Using an Expression Array Targeting the Pig Immune Response. BMC Genom..

[47] Huang T.-H., Uthe J.J., Bearson S.M.D., Demirkale C.Y., Nettleton D., Knetter S., Christian C., Ramer-Tait A.E., Wannemuehler M.J., Tuggle C.K. (2011). Distinct Peripheral Blood RNA Responses to Salmonella in Pigs Differing in Salmonella Shedding Levels: Intersection of IFNG, TLR and miRNA Pathways. PLoS ONE.

[48] Mach N., Gao Y., Lemonnier G., Lecardonnel J., Oswald I.P., Estellé J., Rogel-Gaillard C. (2013). The Peripheral Blood Transcriptome Reflects Variations in Immunity Traits in Swine: Towards the Identification of Biomarkers. BMC Genom..

[49] Clapperton M., Diack A.B., Matika O., Glass E.J., Gladney C.D., Mellencamp M.A., Hoste A., Bishop S.C. (2009). Traits Associated with Innate and Adaptive Immunity in Pigs: Heritability and Associations with Performance under Different Health Status Conditions. Genet. Sel. Evol..

[50] Henryon M., Heegaard P., Nielsen J., Berg P., Juul-Madsen H. (2006). Immunological Traits Have the Potential to Improve Selection of Pigs for Resistance to Clinical and Subclinical Disease. Anim. Sci..

[51] Clapperton M., Glass E.J., Bishop S.C. (2008). Pig Peripheral Blood Mononuclear Leucocyte Subsets Are Heritable and Genetically Correlated with Performance. Anim. Int. J. Anim. Biosci..

[52] Li J., Yu Y.-J., Feng L., Cai X.-B., Tang H.-B., Sun S.-K., Zhang H.-Y., Liang J.-J., Luo T.R. (2010). Global Transcriptional Profiles in Peripheral Blood Mononuclear Cell during Classical Swine Fever Virus Infection. Virus Res..

[53] Feng W.-H., Tompkins M.B., Xu J.-S., Zhang H.-X., McCaw M.B. (2003). Analysis of Constitutive Cytokine Expression by Pigs Infected In-Utero with Porcine Reproductive and Respiratory Syndrome Virus. Vet. Immunol. Immunopathol..

[54] Collins J.E., Benfield D.A., Christianson W.T., Harris L., Hennings J.C., Shaw D.P., Goyal S.M., McCullough S., Morrison R.B., Joo H.S. (1992). Isolation of Swine Infertility and Respiratory Syndrome Virus (Isolate ATCC VR-2332) in North America and Experimental Reproduction of the Disease in Gnotobiotic Pigs. J. Vet. Diagn. Investig..

[55] Xiao S., Mo D., Wang Q., Jia J., Qin L., Yu X., Niu Y., Zhao X., Liu X., Chen Y. (2010). Aberrant Host Immune Response Induced by Highly Virulent PRRSV Identified by Digital Gene Expression Tag Profiling. BMC Genom..

[56] Uddin M.J., Nuro-Gyina P.K., Islam M.A., Tesfaye D., Tholen E., Looft C., Schellander K., Cinar M.U. (2012). Expression Dynamics of Toll-like Receptors mRNA and Cytokines in Porcine Peripheral Blood Mononuclear Cells Stimulated by Bacterial Lipopolysaccharide. Vet. Immunol. Immunopathol..

[57] Kumar H., Kawai T., Akira S. (2011). Pathogen Recognition by the Innate Immune System. Int. Rev. Immunol..

[58] Luo R., Xiao S., Jiang Y., Jin H., Wang D., Liu M., Chen H., Fang L. (2008). Porcine Reproductive and Respiratory Syndrome Virus (PRRSV) Suppresses Interferon-Beta Production by Interfering with the RIG-I Signaling Pathway. Mol. Immunol..

[59] Zhou P., Zhai S., Zhou X., Lin P., Jiang T., Hu X., Jiang Y., Wu B., Zhang Q., Xu X. (2011). Molecular Characterization of Transcriptome-Wide Interactions between Highly Pathogenic Porcine Reproductive and Respiratory Syndrome Virus and Porcine Alveolar Macrophages In Vivo. Int. J. Biol. Sci..

[60] Sun Y., Han M., Kim C., Calvert J.G., Yoo D. (2012). Interplay between Interferon-Mediated Innate Immunity and Porcine Reproductive and Respiratory Syndrome Virus. Viruses.

[61] Kimman T.G., Cornelissen L.A., Moormann R.J., Rebel J.M.J., Stockhofe-Zurwieden N. (2009). Challenges for Porcine Reproductive and Respiratory Syndrome Virus (PRRSV) Vaccinology. Vaccine.

[62] Lunney J.K., Fang Y., Ladinig A., Chen N., Li Y., Rowland B., Renukaradhya G.J. (2016). Porcine Reproductive and Respiratory Syndrome Virus (PRRSV): Pathogenesis and Interaction with the Immune System. Annu. Rev. Anim. Biosci..

[63] Wysocki M., Chen H., Steibel J.P., Kuhar D., Petry D., Bates J., Johnson R., Ernst C.W., Lunney J.K. (2012). Identifying Putative Candidate Genes and Pathways Involved in Immune Responses to Porcine Reproductive and Respiratory Syndrome Virus (PRRSV) Infection. Anim. Genet..

[64] Dwivedi V., Manickam C., Binjawadagi B., Linhares D., Murtaugh M.P., Renukaradhya G.J. (2012). Evaluation of Immune Responses to Porcine Reproductive and Respiratory Syndrome Virus in Pigs During Early Stage of Infection Under Farm Conditions. Virol. J..

[65] Zhang L., Liu J., Bai J., Wang X., Li Y., Jiang P. (2013). Comparative Expression of Toll-like Receptors and Inflammatory Cytokines in Pigs Infected with Different Virulent Porcine Reproductive and Respiratory Syndrome Virus Isolates. Virol. J..

[66] Badaoui B., Rutigliano T., Anselmo A., Vanhee M., Nauwynck H., Giuffra E., Botti S. (2014). RNA-Sequence Analysis of Primary Alveolar Macrophages after In Vitro Infection with Porcine Reproductive and Respiratory Syndrome Virus Strains of Differing Virulence. PLoS ONE.

[67] Xiao S., Jia J., Mo D., Wang Q., Qin L., He Z., Zhao X., Huang Y., Li A., Yu J. (2010). Understanding PRRSV Infection in Porcine Lung Based on Genome-Wide Transcriptome Response Identified by Deep Sequencing. PLoS ONE.

[68] Arceo M.E., Ernst C.W., Lunney J.K., Choi I., Raney N.E., Huang T., Tuggle C.K., Rowland R.R.R., Steibel J.P. (2012). Characterizing Differential Individual Response to Porcine Reproductive and Respiratory Syndrome Virus Infection through Statistical and Functional Analysis of Gene Expression. Front. Genet..

[69] Schroyen M., Eisley C., Koltes J.E., Fritz-Waters E., Choi I., Plastow G.S., Guan L., Stothard P., Bao H., Kommadath A. (2016). Bioinformatic Analyses in Early Host Response to Porcine Reproductive and Respiratory Syndrome Virus (PRRSV) Reveals Pathway Differences between Pigs with Alternate Genotypes for a Major Host Response QTL. BMC Genom..

[70] Schroyen M., Steibel J.P., Koltes J.E., Choi I., Raney N.E., Eisley C., Fritz-Waters E., Reecy J.M., Dekkers J.C.M., Rowland R.R.R. (2015). Whole Blood Microarray Analysis of Pigs Showing Extreme Phenotypes after a Porcine Reproductive and Respiratory Syndrome Virus Infection. BMC Genom..

[71] Zhuge Z.-Y., Zhu Y.-H., Liu P.-Q., Yan X.-D., Yue Y., Weng X.-G., Zhang R., Wang J.-F. (2012). Effects of Astragalus Polysaccharide on Immune Responses of Porcine PBMC Stimulated with PRRSV or CSFV. PLoS ONE.

[72] Ait-Ali T., Díaz I., Soldevila F., Cano E., Li Y., Wilson A.D., Giotti B., Archibald A.L., Mateu E., Darwich L. (2016). Distinct Functional Enrichment of Transcriptional Signatures in Pigs with High and Low IFN-Gamma Responses after Vaccination with a Porcine Reproductive and Respiratory Syndrome Virus (PRRSV). Vet. Res..

[73] Islam M.A., Große-Brinkhaus C., Pröll M.J., Uddin M.J., Aqter Rony S., Tesfaye D., Tholen E., Hoelker M., Schellander K., Neuhoff C. (2017). PBMC Transcriptome Profiles Identifies Potential Candidate Genes and Functional Networks Controlling the Innate and the Adaptive Immune Response to PRRSV Vaccine in Pietrain Pig. PLoS ONE.

[74] Jiang B., Li L., Wu Y., Wang X., Gao N., Xu Z., Guo C., He S., Zhang G., Chen Y. (2024). Unveiling Shared Immune Responses in Porcine Alveolar Macrophages during ASFV and PRRSV Infection Using Single-Cell RNA-Seq. Microorganisms.

[75] Ait-Ali T., Wilson A.D., Carré W., Westcott D.G., Frossard J.-P., Mellencamp M.A., Mouzaki D., Matika O., Waddington D., Drew T.W. (2011). Host Inhibits Replication of European Porcine Reproductive and Respiratory Syndrome Virus in Macrophages by Altering Differential Regulation of Type-I Interferon Transcriptional Response. Immunogenetics.

[76] Bates J.S., Petry D.B., Eudy J., Bough L., Johnson R.K. (2008). Differential Expression in Lung and Bronchial Lymph Node of Pigs with High and Low Responses to Infection with Porcine Reproductive and Respiratory Syndrome Virus. J. Anim. Sci..

[77] Ait-Ali T., Wilson A.W., Finlayson H., Carré W., Ramaiahgari S.C., Westcott D.G., Waterfall M., Frossard J.-P., Baek K.-H., Drew T.W. (2009). Functional Analysis of the Porcine USP18 and Its Role during Porcine Arterivirus Replication. Gene.

[78] Genini S., Delputte P.L., Malinverni R., Cecere M., Stella A., Nauwynck H.J., Giuffra E. (2008). Genome-Wide Transcriptional Response of Primary Alveolar Macrophages Following Infection with Porcine Reproductive and Respiratory Syndrome Virus. J. Gen. Virol..

[79] Auray G., Lachance C., Wang Y., Gagnon C.A., Segura M., Gottschalk M. (2016). Transcriptional Analysis of PRRSV-Infected Porcine Dendritic Cell Response to *Streptococcus suis* Infection Reveals Up-Regulation of Inflammatory-Related Genes Expression. PLoS ONE.

[80] Walker K., Hong L., Reecy J.M., Fritz-Waters E., Dong Q., Rowland R.R.R., Ireland D.D.C., Verthelyi D., Tuggle C.K., Dekkers J.C.M. (2017). Probing Host Immune Response to Porcine Reproductive and Respiratory Syndrome Virus (PRRSV) Infection Using 3′RNAseq and NanoString Arrays. J. Immunol..

[81] Doeschl-Wilson A.B., Villanueva B., Kyriazakis I. (2012). The First Step toward Genetic Selection for Host Tolerance to Infectious Pathogens: Obtaining the Tolerance Phenotype through Group Estimates. Front. Genet..

[82] Råberg L., Graham A.L., Read A.F. (2009). Decomposing Health: Tolerance and Resistance to Parasites in Animals. Philos. Trans. R. Soc. Lond. B Biol. Sci..

[83] Medzhitov R., Schneider D.S., Soares M.P. (2012). Disease Tolerance as a Defense Strategy. Science.

[84] Lewis C.R.G., Ait-Ali T., Clapperton M., Archibald A.L., Bishop S. (2007). Genetic Perspectives on Host Responses to Porcine Reproductive and Respiratory Syndrome (PRRS). Viral Immunol..

[85] Guy S.Z.Y., Thomson P.C., Hermesch S. (2012). Selection of Pigs for Improved Coping with Health and Environmental Challenges: Breeding for Resistance or Tolerance?. Front. Genet..

[86] Jenner R.G., Young R.A. (2005). Insights into Host Responses against Pathogens from Transcriptional Profiling. Nat. Rev. Microbiol..

[87] Zhang X., Shin J., Molitor T.W., Schook L.B., Rutherford M.S. (1999). Molecular Responses of Macrophages to Porcine Reproductive and Respiratory Syndrome Virus Infection. Virology.

[88] Hu Z.-L., Reecy J.M. (2007). Animal QTLdb: Beyond a Repository. A Public Platform for QTL Comparisons and Integration with Diverse Types of Structural Genomic Information. Mamm. Genome.

[89] Uddin M.J., Große-Brinkhaus C., Cinar M.U., Jonas E., Tesfaye D., Tholen E., Juengst H., Looft C., Ponsuksili S., Wimmers K. (2010). Mapping of Quantitative Trait Loci for Mycoplasma and Tetanus Antibodies and Interferon-Gamma in a Porcine F_2_ Duroc x Pietrain Resource Population. Mamm. Genome.

[90] Boddicker N., Waide E.H., Rowland R.R.R., Lunney J.K., Garrick D.J., Reecy J.M., Dekkers J.C.M. (2012). Evidence for a Major QTL Associated with Host Response to Porcine Reproductive and Respiratory Syndrome Virus Challenge. J. Anim. Sci..

[91] Boddicker N.J., Bjorkquist A., Rowland R.R.R., Lunney J.K., Reecy J.M., Dekkers J.C.M. (2014). Genome-Wide Association and Genomic Prediction for Host Response to Porcine Reproductive and Respiratory Syndrome Virus Infection. Genet. Sel. Evol..

[92] Koltes J.E., Fritz-Waters E., Eisley C.J., Choi I., Bao H., Kommadath A., Serão N.V.L., Boddicker N.J., Abrams S.M., Schroyen M. (2015). Identification of a Putative Quantitative Trait Nucleotide in Guanylate Binding Protein 5 for Host Response to PRRS Virus Infection. BMC Genom..

[93] Abella G., Pena R.N., Nogareda C., Armengol R., Vidal A., Moradell L., Tarancon V., Novell E., Estany J., Fraile L. (2016). A WUR SNP Is Associated with European Porcine Reproductive and Respiratory Virus Syndrome Resistance and Growth Performance in Pigs. Res. Vet. Sci..

[94] Li Y., Sun Y., Xing F., Kang L., Wang P., Wang L., Liu H., Li Y., Jiang Y. (2014). Identification of a Single Nucleotide Promoter Polymorphism Regulating the Transcription of Ubiquitin Specific Protease 18 Gene Related to the Resistance to Porcine Reproductive and Respiratory Syndrome Virus Infection. Vet. Immunol. Immunopathol..

[95] Medzhitov R. (2009). Damage Control in Host-Pathogen Interactions. Proc. Natl. Acad. Sci. USA.

[96] Gibbons R.A., Sellwood R., Burrows M., Hunter P.A. (1977). Inheritance of Resistance to Neonatal *E. coli* Diarrhoea in the Pig: Examination of the Genetic System. Theor. Appl. Genet..

[97] Van Gorp H., Van Breedam W., Delputte P.L., Nauwynck H.J. (2009). The Porcine Reproductive and Respiratory Syndrome Virus Requires Trafficking through CD163-Positive Early Endosomes, but Not Late Endosomes, for Productive Infection. Arch. Virol..

[98] Calvert J.G., Slade D.E., Shields S.L., Jolie R., Mannan R.M., Ankenbauer R.G., Welch S.-K.W. (2007). CD163 Expression Confers Susceptibility to Porcine Reproductive and Respiratory Syndrome Viruses. J. Virol..

[99] Duan X., Nauwynck H.J., Pensaert M.B. (1997). Effects of Origin and State of Differentiation and Activation of Monocytes/Macrophages on Their Susceptibility to Porcine Reproductive and Respiratory Syndrome Virus (PRRSV). Arch. Virol..

[100] Shanmukhappa K., Kim J.-K., Kapil S. (2007). Role of CD151, A Tetraspanin, in Porcine Reproductive and Respiratory Syndrome Virus Infection. Virol. J..

[101] Jusa E.R., Inaba Y., Kouno M., Hirose O. (1997). Effect of Heparin on Infection of Cells by Porcine Reproductive and Respiratory Syndrome Virus. Am. J. Vet. Res..

[102] Kim J.-K., Fahad A.-M., Shanmukhappa K., Kapil S. (2006). Defining the Cellular Target(s) of Porcine Reproductive and Respiratory Syndrome Virus Blocking Monoclonal Antibody 7G10. J. Virol..

[103] Huang Y.W., Dryman B.A., Li W., Meng X.J. (2009). Porcine DC-SIGN: Molecular Cloning, Gene Structure, Tissue Distribution and Binding Characteristics. Dev. Comp. Immunol..

[104] Zhang Q., Yoo D. (2015). PRRS Virus Receptors and Their Role for Pathogenesis. Vet. Microbiol..

[105] Delputte P.L., Costers S., Nauwynck H.J. (2005). Analysis of Porcine Reproductive and Respiratory Syndrome Virus Attachment and Internalization: Distinctive Roles for Heparan Sulphate and Sialoadhesin. J. Gen. Virol..

[106] Duan X., Nauwynck H.J., Favoreel H.W., Pensaert M.B. (1998). Identification of a Putative Receptor for Porcine Reproductive and Respiratory Syndrome Virus on Porcine Alveolar Macrophages. J. Virol..

[107] Van Gorp H., Van Breedam W., Delputte P.L., Nauwynck H.J. (2008). Sialoadhesin and CD163 Join Forces during Entry of the Porcine Reproductive and Respiratory Syndrome Virus. J. Gen. Virol..

[108] Hinton A., Bond S., Forgac M. (2009). V-ATPase Functions in Normal and Disease Processes. Pflüg. Arch..

[109] Whitworth K.M., Rowland R.R.R., Ewen C.L., Trible B.R., Kerrigan M.A., Cino-Ozuna A.G., Samuel M.S., Lightner J.E., McLaren D.G., Mileham A.J. (2016). Gene-Edited Pigs Are Protected from Porcine Reproductive and Respiratory Syndrome Virus. Nat. Biotechnol..

[110] Burkard C., Lillico S.G., Reid E., Jackson B., Mileham A.J., Ait-Ali T., Whitelaw C.B.A., Archibald A.L. (2017). Precision Engineering for PRRSV Resistance in Pigs: Macrophages from Genome Edited Pigs Lacking CD163 SRCR5 Domain Are Fully Resistant to Both PRRSV Genotypes While Maintaining Biological Function. PLoS Pathog..

[111] Nesbitt C., Galina Pantoja L., Beaton B., Chen C.-Y., Culbertson M., Harms P., Holl J., Sosnicki A., Reddy S., Rotolo M. (2024). Pigs Lacking the SRCR5 Domain of CD163 Protein Demonstrate Heritable Resistance to the PRRS Virus and No Changes in Animal Performance from Birth to Maturity. Front. Genome Ed..

[112] Liu Y., Yang L., Xiang H.-Y., Niu M., Deng J.-C., Li X.-Y., Hao W.-J., Ou-Yang H.-S., Liu T.-Y., Tang X.-C. (2024). Genetically Modified Pigs with CD163 Point Mutation Are Resistant to HP-PRRSV Infection. Zool. Res..

[113] Hung S.-W., Chuang C.-K., Wong C.-H., Yen C.-H., Peng S.-H., Yang C., Chen M.-C., Yang T.-S., Tu C.-F. (2023). Activated Macrophages of CD 163 Gene Edited Pigs Generated by Direct Cytoplasmic Microinjection with CRISPR gRNA/Cas9 mRNA Are Resistant to PRRS Virus Assault. Anim. Biotechnol..

[114] Wu C., Wang H., Zhang W., Cheng M., Wang Y., Chen L., Tang C., Dai Y., Zhang L. (2026). Pigs with CD163 Mutation Conferred PRRSV Resistance. Animals.

[115] US-FDA (US Food and Drug Administration), Center for Veterinary Medicine (2025). Freedom of Information Summary: Intentional Genomic Alteration in CD163Δexon7 Line Pigs Conferring Resistance to Porcine Reproductive and Respiratory Syndrome Virus. https://animaldrugsatfda.fda.gov/adafda/app/search/public/document/downloadFoi/16887.

[116] Islam M.A., Neuhoff C., Aqter Rony S., Große-Brinkhaus C., Uddin M.J., Hölker M., Tesfaye D., Tholen E., Schellander K., Pröll-Cornelissen M.J. (2019). PBMCs Transcriptome Profiles Identified Breed-Specific Transcriptome Signatures for PRRSV Vaccination in German Landrace and Pietrain Pigs. PLoS ONE.

[117] Lough G., Hess A., Hess M., Rashidi H., Matika O., Lunney J.K., Rowland R.R.R., Kyriazakis I., Mulder H.A., Dekkers J.C.M. (2018). Harnessing Longitudinal Information to Identify Genetic Variation in Tolerance of Pigs to Porcine Reproductive and Respiratory Syndrome Virus Infection. Genet. Sel. Evol..

[118] Huang G., Liu X., Tang X., Du L., Feng W., Hu X., Zhu L., Li Q., Suo X. (2017). Increased Neutralizing Antibody Production and Interferon-γ Secretion in Response to Porcine Reproductive and Respiratory Syndrome Virus Immunization in Genetically Modified Pigs. Front. Immunol..

[119] Cigan A.M., Knap P.W. (2022). Technical Considerations towards Commercialization of Porcine Respiratory and Reproductive Syndrome (PRRS) Virus Resistant Pigs. CABI Agric. Biosci..

[120] Wells K.D., Bardot R., Whitworth K.M., Trible B.R., Fang Y., Mileham A., Kerrigan M.A., Samuel M.S., Prather R.S., Rowland R.R.R. (2017). Replacement of Porcine CD163 Scavenger Receptor Cysteine-Rich Domain 5 with a CD163-Like Homolog Confers Resistance of Pigs to Genotype 1 but Not Genotype 2 Porcine Reproductive and Respiratory Syndrome Virus. J. Virol..

[121] Popescu L., Gaudreault N.N., Whitworth K.M., Murgia M.V., Nietfeld J.C., Mileham A., Samuel M., Wells K.D., Prather R.S., Rowland R.R.R. (2017). Genetically Edited Pigs Lacking CD163 Show No Resistance Following Infection with the African Swine Fever Virus Isolate, Georgia 2007/1. Virology.

[122] Bisimwa P.N., Ongus J.R., Tonui R., Bisimwa E.B., Steinaa L. (2024). Resistance to African Swine Fever Virus among African Domestic Pigs Appears to Be Associated with a Distinct Polymorphic Signature in the RelA Gene and Upregulation of RelA Transcription. Virol. J..

[123] Simms E. (2000). Defining Tolerance as a Norm of Reaction. Evol. Ecol..

[124] Wilkie B., Mallard B. (1999). Selection for High Immune Response: An Alternative Approach to Animal Health Maintenance?. Vet. Immunol. Immunopathol..

[125] Zhao S., Zhu M., Chen H. (2012). Immunogenomics for Identification of Disease Resistance Genes in Pigs: A Review Focusing on Gram-Negative Bacilli. J. Anim. Sci. Biotechnol..

[126] Zhu M., Zhao S. (2007). Candidate Gene Identification Approach: Progress and Challenges. Int. J. Biol. Sci..

[127] Schroyen M., Tuggle C.K. (2015). Current Transcriptomics in Pig Immunity Research. Mamm. Genome.

[128] Tuggle C.K., Bearson S.M.D., Uthe J.J., Huang T.H., Couture O.P., Wang Y.F., Kuhar D., Lunney J.K., Honavar V. (2010). Methods for Transcriptomic Analyses of the Porcine Host Immune Response: Application to Salmonella Infection Using Microarrays. Vet. Immunol. Immunopathol..

[129] Miller L.C., Harhay G.P., Lager K.M., Smith T.P.L., Neill J.D. (2008). Effect of Porcine Reproductive and Respiratory Syndrome Virus on Porcine Alveolar Macrophage Function as Determined Using Serial Analysis of Gene Expression (SAGE). Dev. Biol..

[130] Reiner G., Willems H., Pesch S., Ohlinger V.F. (2010). Variation in Resistance to the Porcine Reproductive and Respiratory Syndrome Virus (PRRSV) in Pietrain and Miniature Pigs. J. Anim. Breed. Genet..

[131] Xing J., Xing F., Zhang C., Zhang Y., Wang N., Li Y., Yang L., Jiang C., Zhang C., Wen C. (2014). Genome-Wide Gene Expression Profiles in Lung Tissues of Pig Breeds Differing in Resistance to Porcine Reproductive and Respiratory Syndrome Virus. PLoS ONE.

[132] Lunney J.K., Chen H. (2010). Genetic Control of Host Resistance to Porcine Reproductive and Respiratory Syndrome Virus (PRRSV) Infection. Virus Res..

[133] Wong L., Jiang K., Chen Y., Hennon T., Holmes L., Wallace C.A., Jarvis J.N. (2016). Limits of Peripheral Blood Mononuclear Cells for Gene Expression-Based Biomarkers in Juvenile Idiopathic Arthritis. Sci. Rep..

[134] Palmer C., Diehn M., Alizadeh A.A., Brown P.O. (2006). Cell-Type Specific Gene Expression Profiles of Leukocytes in Human Peripheral Blood. BMC Genom..

[135] Osman A., Hitzler W.E., Ameur A., Provost P. (2015). Differential Expression Analysis by RNA-Seq Reveals Perturbations in the Platelet mRNA Transcriptome Triggered by Pathogen Reduction Systems. PLoS ONE.

[136] Fairbairn L., Kapetanovic R., Beraldi D., Sester D.P., Tuggle C.K., Archibald A.L., Hume D.A. (2013). Comparative Analysis of Monocyte Subsets in the Pig. J. Immunol..

[137] Steuerman Y., Gat-Viks I. (2016). Exploiting Gene-Expression Deconvolution to Probe the Genetics of the Immune System. PLoS Comput. Biol..

[138] Shoemaker J.E., Lopes T.J.S., Ghosh S., Matsuoka Y., Kawaoka Y., Kitano H. (2012). CTen: A Web-Based Platform for Identifying Enriched Cell Types from Heterogeneous Microarray Data. BMC Genom..

[139] Zhao Y., Simon R. (2010). Gene Expression Deconvolution in Clinical Samples. Genome Med..

[140] Venet D., Pecasse F., Maenhaut C., Bersini H. (2001). Separation of Samples into Their Constituents Using Gene Expression Data. Bioinformatics.

[141] Wiarda J.E., Kapoor M., Sivasankaran S.K., Byrne K.A., Loving C.L., Tuggle C.K. (2026). A Single-Cell Immune Atlas of Primary and Secondary Lymphoid Organs in Pigs. Front. Immunol..

