# Supplementary material for: Peripheral Blood Mononuclear Cell Transcriptomics in Porcine Reproductive and Respiratory Syndrome: A Window into Innate Immune Resistance and Tolerance to Viral Disease
Source: Viruses. 2026 Sep 9;18(9):994. doi: 10.3390/v18090994 (PMC13612018; doi:10.3390/v18090994)
Supplement: Supplementary file 1 [file viruses-18-00994-s001.zip › viruses-4537454-supplementary.pdf]

Supplementary Table S1. Concept blocks and Boolean search combinations used for literature retrieval

Searches were conducted primarily in PubMed/MEDLINE and supplemented by Scopus, Web of Science, Google Scholar, FAANG, CABI resources, the U.S. FDA Center for Veterinary Medicine, and selective bioRxiv searching. Terms within each concept block were combined with OR, and concept blocks were combined with AND. Searches were run through July 2026 without a lower date restriction.

Concept block	Representative search terms / keywords	Example Boolean combination	Purpose / evidence targeted
**1. Sample / cell type**	"peripheral blood mononuclear cell*" OR PBMC* OR "whole blood" OR leukocyte* OR monocyte* OR lymphocyte*	(PBMC* OR "peripheral blood mononuclear cell*" OR "whole blood") AND PRRSV AND (transcriptom* OR "RNA-seq" OR microarray)	Identify blood- and PBMC-based transcriptomic studies relevant to systemic and innate immune responses.
**2. Pathogen / disease**	"porcine reproductive and respiratory syndrome" OR PRRS OR PRRSV OR arterivirus OR "porcine reproductive and respiratory syndrome virus"	(PBMC* OR "whole blood") AND (PRRS OR PRRSV OR arterivirus) AND transcriptom*	Retrieve studies of host–PRRSV interactions, vaccination, infection, pathogenesis, and molecular response.
**3. Host-response phenotype**	resistance OR tolerance OR resilience OR susceptibility OR immunocompetence OR "immune competence" OR "host response" OR QTL OR "quantitative trait locus/loci" OR "candidate gene*"	(PRRS OR PRRSV) AND (resistance OR tolerance OR immunocompetence OR susceptibility) AND (gene* OR QTL OR transcriptom*)	Capture molecular-genetic evidence for resistance, tolerance, susceptibility, immunocompetence, candidate genes, and QTL.
**4. Transcriptomic technology**	transcriptom* OR "gene expression" OR "RNA sequencing" OR RNA-seq OR microarray OR "single-cell RNA-seq" OR scRNA-seq OR deconvolution OR "cell-type enrichment"	(PBMC* OR "whole blood") AND (PRRS OR PRRSV) AND (transcriptom* OR RNA-seq OR microarray OR "single-cell" OR deconvolution)	Identify bulk and single-cell transcriptome studies and methods for resolving PBMC heterogeneity.
**5. Innate immune signaling / host–virus interaction**	"innate immunity" OR "pattern recognition receptor*" OR PRR OR TLR OR "Toll-like receptor*" OR RIG-I OR RLR OR IRF3 OR IRF7 OR NF-kB OR MAPK OR interferon* OR JAK-STAT OR ISG*	(PRRSV OR arterivirus) AND (PBMC* OR macrophage*) AND ("innate immunity" OR TLR OR RIG-I OR interferon* OR JAK-STAT OR ISG*)	Retrieve mechanistic evidence on conserved antiviral sensing and signaling pathways relevant to porcine PBMC responses.
**6. Tissue / cell models for PRRSV**	lung OR "bronchial lymph node" OR "pulmonary alveolar macrophage*" OR PAM OR "uterine endotheli*" OR "dendritic cell*" OR "whole blood" OR PBMC*	(PRRS OR PRRSV) AND (lung OR "alveolar macrophage*" OR "dendritic cell*" OR "whole blood" OR PBMC*) AND transcriptom*	Compare PBMCs with respiratory and other tissue/cell models used to study the host–PRRSV transcriptome.
**7. Genetic resistance candidates / viral entry**	CD163 OR CD169 OR Siglec-1 OR sialoadhesin OR SRCR OR CD151 OR "heparan sulfate" OR vimentin OR CD209 OR ATP6V1B2 OR MX1 OR USP18 OR GBP5 OR GBP1 OR ADAM17 OR CD28 OR SSC4 OR WUR	(PRRS OR PRRSV) AND (CD163 OR GBP5 OR USP18 OR ADAM17 OR SSC4 OR WUR) AND (resistance OR susceptibility OR "host response")	Retrieve evidence for receptors, antiviral genes, and loci associated with PRRSV entry, replication, resistance, or tolerance.
**8. Vaccination / challenge phenotyping**	vaccin* OR immuniz* OR "live attenuated" OR "modified live vaccine" OR MLV OR challenge OR infection OR viremia OR "viral load" OR "weight gain" OR performance	(PRRSV AND vaccin*) AND (PBMC* OR "whole blood") AND (transcriptom* OR "gene expression") AND (resistance OR tolerance OR immunocompetence)	Capture direct infection/challenge and indirect vaccination models used to phenotype host response over time.
**9. Breed / genetic variation**	breed* OR genotype* OR "genetic variation" OR Landrace OR "German Landrace" OR Pietrain OR Duroc OR Hampshire OR "Large White"	(PRRS OR PRRSV) AND (Landrace OR Pietrain OR Duroc OR breed*) AND (transcriptom* OR "gene expression")	Identify breed-dependent and genetically determined variation in

			PRRSV-induced transcriptional and immune responses.
**10. Translational biotechnology / gene editing**	CD163 OR "gene edit*" OR CRISPR OR CRISPR-Cas9 OR knockout OR "exon 7" OR SRCR5 OR commerciali* OR approval OR FDA OR "gene-edited pig*"	(PRRSV OR PRRS) AND (CD163 AND ("gene edit*" OR CRISPR OR knockout OR SRCR5 OR "exon 7"))	Trace translation of mechanistic resistance candidates into gene-edited PRRSV-resistant pigs and regulatory/commercial milestones.
**11. PBMC technical quality / pre-analytics**	PBMC AND (isolation OR Ficoll OR anticoagulant OR cryopreservation OR storage OR "sample processing" OR "RNA quality" OR "reference gene*" OR normalization)	PBMC* AND transcriptom* AND (isolation OR cryopreservation OR storage OR normalization OR "reference gene*")	Retrieve evidence on sample handling, storage, cell isolation, RNA quality, normalization, and reproducibility of PBMC transcriptomes.
**12. PBMC annotation / atlases / deconvolution**	FAANG OR "Functional Annotation of Animal Genomes" OR "pig immune atlas" OR "swine transcriptome atlas" OR "single-cell atlas" OR CIBERSORTx OR deconvolution OR "sorted PBMC*"	(pig OR porcine OR swine) AND (PBMC* OR blood) AND (atlas OR FAANG OR deconvolution OR CIBERSORTx OR "single-cell")	Capture recent annotation resources and computational/single-cell approaches that improve interpretation of heterogeneous PBMC data.

Notes: The table provides representative, manuscript-aligned search syntax rather than a single fixed systematic-review query. Wildcard (*) indicates truncation where supported by the database. Database-specific field tags and syntax can be adapted as needed. Backward reference screening and forward citation chaining were additionally used to identify foundational and newer studies.
